# Low excretor glutaric aciduria type 1 of insidious onset with dystonia and atypical clinical features, a diagnostic dilemma

**DOI:** 10.1002/jmd2.12187

**Published:** 2020-11-16

**Authors:** Jason Foran, Michael Moore, Ellen Crushell, Ina Knerr, Niamh McSweeney

**Affiliations:** ^1^ Department of Paediatric Neurology Cork University Hospital Cork Republic of Ireland; ^2^ Department of Radiology Cork University Hospital Cork Republic of Ireland; ^3^ National Centre for Inherited Metabolic Disorders Children's Health Ireland at Temple Street Dublin Republic of Ireland

**Keywords:** dystonia, glutaric aciduria type 1, low excretor, organic acidurias

## Abstract

A 4‐year‐old girl was referred for reassessment of dyskinetic cerebral palsy. Initial investigations in her country of birth, India, had not yielded a diagnosis. MRI brain in infancy revealed bilateral putamen hyperintensity. She had generalized dyskinesia predominantly bulbar and limbs. Motor and speech development were most affected with preservation of cognitive development. There was no history of acute encephalopathic crisis or status dystonicus. Initial urine organic acids and amino acids and acylcarnitine profile (ACP) were normal. A dystonia genetic panel showed compound heterozygosity with a pathogenic variant and a variant of uncertain significance in the *GCDH* gene. The latter is hitherto undescribed and is indicative of a potential diagnosis of glutaric aciduria type 1 (alternatively glutaric acidemia type 1) (GA‐1), an autosomal recessive disorder of mitochondrial lysine/hydroxylysine and tryptophan metabolism. Repeat urine organic acids showed isolated slightly increased 3‐hydroxy glutarate excretion consistent with GA‐1 and characterizing the patient as a “low excretor,” a diagnostic sub‐group where diagnosis is more challenging but prognosis is similar. Repeat MRI Brain at age 4 showed volume loss and symmetric T2 hyperintensity in the posterior putamina bilaterally. This case highlights the diagnostic dilemma of GA‐1 where differing clinical courses, genetic variants, neuroradiological findings, and biochemical excretion patterns may lead to a later diagnosis. The presence of newborn screening for GA‐1 should not dull the clinician's suspicion of the possibility that GA‐1 may present with a complex movement disorder. Timely diagnosis and treatment is essential, as neurological sequelae are largely irreversible.

**Synopsis**

A case showing glutaric aciduria type 1 can present insidiously and atypically and a high index of suspicion should be kept in dystonic patients even outside the typical populations to avoid delayed treatment.

## 1 CASE REPORT

A 4‐year‐old girl was referred for assessment of a 3‐year diagnosis of dyskinetic cerebral palsy. Specifically these related to limb and oromotor dyskinesia with increased drooling. She had initially been investigated in India prior to moving to Ireland. She was the first child of nonconsanguineous parents from India. The pregnancy was notable for polyhydramnios but fetal movements were normal as was an anomaly scan. She was born at term and had poor feeding in the neonatal period with both breast and formula feeding. Solids were introduced normally at 6 months without any dysphagia. Developmentally she showed delay with a social smile and eye contact from 3 months, she rolled at 6 months with good head control from 8 months. She sat with support at 18 months and without support at 2 years. She was able to stand with support from 2 ½ years and could walk with both hands held, at presentation. Expressive speech was delayed but cognition was reportedly intact albeit not formally assessed. There was no history of any acute encephalopathy precipitated by intercurrent illness or indeed regression of skills.

On examination, her height and weight were both on the 50th centile. Her OFC measured 49.8 cm (11th centile), at 3 years of age, her OFC was 48 cm (25th centile), birth OFC was not available. She had normal eye movements and no telangiectasia was present. The remainder of her cranial nerve examination was normal. She had significant oromotor dyskinesia with marked drooling. She had increased peripheral tone in all four limbs, bilateral upgoing plantars, and tight Achilles tendons. There was left sided weakness with sensory neglect.

Initial investigations performed in India were normal including TFTs, LFTs, FBC, CPK, amino acids, acylcarnitine as well as free and total carnitine, urinary GAGs and organic acids. An MRI brain there showed bilateral putamen hyperintensity.

**FIGURE 1:**
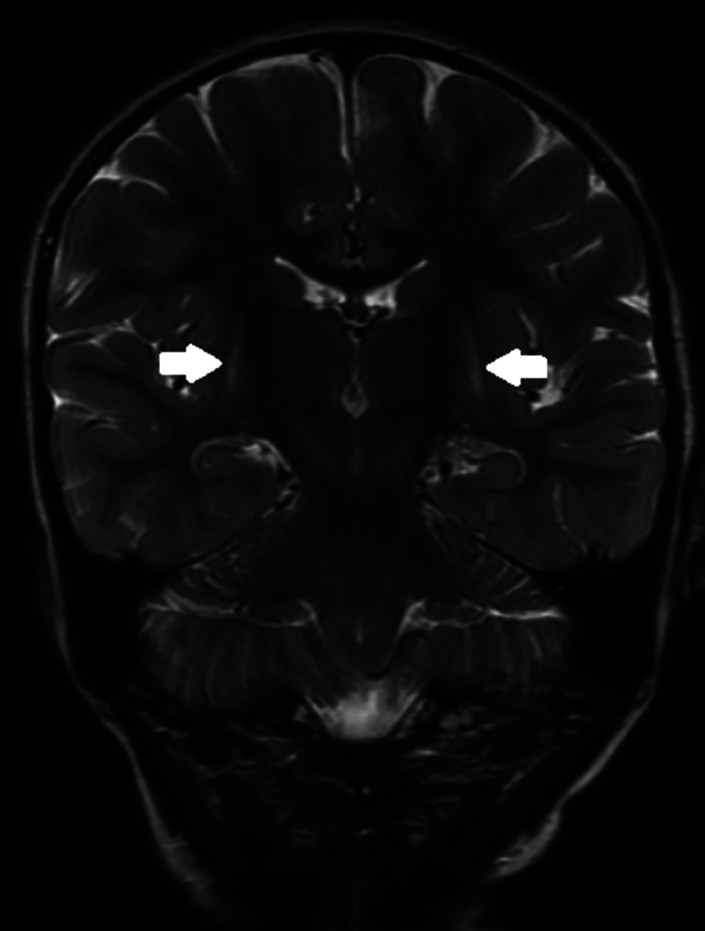
MRI T2 imaging showing subtle posterior putamen hyperintensity (white arrows)

Extensive blood, urine, and CSF metabolic investigations were normal; in particular, she had normal paired CSF and plasma amino acids, lactate, glucose, and CSF protein and neurotransmitters. Amino acid and ACP remained normal with free carnitine of 30.0 μmol/L (reference range 15.5‐46.7) and blood C5DC within normal range. Repeat MRI brain at 4 years and 4 months of age showed volume loss and T2 hyperintensity in the posterior putamina bilaterally and symmetrically (Figure 1).

Genetic testing included a normal microarray and a dystonia gene panel, which showed two variants in the *GCDH* gene:

1. *GCDH* variant c.281G > A;pArg94Gln (het.), NM_000159.3.
2. *GCDH* variant c.1082 + 2 T > G;p.? (het.) NM_000159.3.

Variant 1, a missense variant has been described in two mild GA‐1 phenotypes in Indian family members with extra pyramidal symptoms, abnormal radiology with partly elevated glutaryl carnitine but normal excretion of glutaric acid (<10 mM/mol creatinine).^1^ Variant 2 is unidentified in the literature and is therefore a variant of uncertain significance (VUS) prompting further investigation of a potential GA‐1. Variant 2 is in an essential splice site and was interpreted as potentially having a deleterious effect on splicing. Parental genetic analysis showed paternal possession of variant 1 and maternal possession of variant 2. The dystonia panel was performed by CeGaT GmbH genetics laboratory in Tübingen, Germany. There are 44 target genes for this panel and are selected for causes of both pediatric and adult onset dystonia. Contained in Table 1 is a list of the 44 genes, cross‐referenced with NIH Genetics Home reference to indicate the related diseases. Two other variants of unknown significance were reported, both variants were heterozygous, these were VPS13A c.7078G > A; p.Glu2360Lys, NM_033305.2 which is potentially related to choreoacanthocytosis and ADCY c.3183G > A; p=, which is a silent protein change in a gene associated with familial dyskinesia with facial myokymia and benign hereditary chorea. In silico predictions showed an inconsistent and absent splice effect, respectively. Therefore, further investigation was focused on GA‐1. The genome build was human reference genome (hg19) to which sequence reads were mapped. Only variants with a minor allele frequency (MAF) < 1.5% were evaluated, known disease‐causing variants (as defined by the Human Genome Mutation Database) were evaluated up to MAF <5%.

**TABLE 1:** Dystonia panel target genes and associated pathology

ADAR	Aicardi‐Goutières syndrome
ADCY5	*ADCY5*‐related dyskinesia
ANO3	Dystonia 24
ATM	Ataxia‐telangiectasia
ATP1A2	Familial hemiplegic migraine type 2, alternating hemiplegia of childhood, sporadic hemiplegic migraine
ATP1A3	Alternating hemiplegia of childhood
ATP7B	Wilson disease
BCAP31	Chromosome Xq28 deletion syndrome
CACNA1A	Sporadic hemiplegic migraine, 19p13.13 deletion syndrome, episodic ataxia type 2, spinocerebellar ataxia type 6
CIZ1	Adult onset primary cervical dystonia
COL6A3	Limb‐girdle muscular dystrophy, collagen VI‐related myopathy
DNAJC12	Hyperphenylalaninemia (mild, non‐bh4‐deficient)
FA2H	Fatty acid hydroxylase‐associated neurodegeneration
FTL	Neuroferritinopathy
GCDH	Glutaric acidemia type 1
GCH1	Dopa‐responsive dystonia
GNAL	Dystonia 25
HPCA	Torsion dystonia 2
IRF2BPL	Neurodevelopmental disorder with regression, abnormal movements, loss of speech, and seizures
KCNMA1	Paroxysmal nonkinesigenic dyskinesia, 3, with or without generalized epilepsy
KMT2B	Dystonia 28, childhood‐onset
MECR	Dystonia, childhood‐onset, with optic atrophy and basal ganglia abnormalities
NOTCH3	Cerebral autosomal dominant arteriopathy with subcortical infarcts and leukoencephalopathy (CADASIL)
PANK2	Pantothenate kinase‐associated neurodegeneration
PLA2G6	*PLA2G6*‐related dystonia‐parkinsonism, infantile neuroaxonal dystrophy
PNKD	Familial paroxysmal nonkinesigenic dyskinesia
POLG	Alpers‐Huttenlocher syndrome, ataxia neuropathy spectrum, childhood myocerebrohepatopathy syndrome, myoclonic epilepsy myopathy sensory ataxia, Leigh syndrome
PRKRA	Dystonia 16
PRRT2	Familial paroxysmal kinesigenic dyskinesia, familial hemiplegic migraine, infantile convulsions and choreoathetosis
SCN1A	Familial hemiplegic migraine type 3, Lennox‐Gastaut syndrome, malignant migrating partial seizures of infancy
SCN8A	*SCN8A*‐related epilepsy with encephalopathy, Lennox‐Gastaut syndrome, benign infantile seizures
SGCE	Myoclonus‐dystonia
SLC19A3	Biotin‐thiamine‐responsive basal ganglia disease, Leigh syndrome
SLC2A1	GLUT1 deficiency syndrome
SLC30A10	Hypermanganesemia with dystonia 1
SLC39A14	Hypermanganesemia with dystonia 2
SLC6A3	Dopamine transporter deficiency syndrome
SPR	Dopa‐responsive dystonia, sepiapterin reductase deficiency
TH	Tyrosine hydroxylase (TH) deficiency, dopa‐responsive dystonia
THAP1	Dystonia 6
TOR1A	Early‐onset primary dystonia
TUBB4A	*TUBB4A*‐related leukodystrophy, dystonia 4
VAC14	Striatonigral degeneration, childhood‐onset
VPS13A	Chorea‐acanthocytosis

Tandem mass spectrometric analysis of urine acylcarnitine was performed in light of her molecular genetic diagnosis. Before commencement of oral carnitine supplementation, urinary free carnitine was found reduced at 1.99 μmol/mmol creatinine (reference range 2.45 and 125.5 μmol/mmol creatinine), indicative of carnitine depletion, with a raised urinary C5DC (glutarylcarnitine) at 6.8 μmol/mmol creatinine (reference range <cut‐off, <1.7 μmol/mmol creatinine). After 2 days of carnitine supplementation (100 mg/kg per day in two divided doses), urinary free carnitine increased massively (411 μmol/mmol) whereas urinary C5DC increased only marginally (8.2 μmol/mmol creatinine), presumably due to increased carnitine availability, though this may be within biological variation or error of measurement, and overall in keeping with a low excretor phenotype. A repeat urinary organic acid profile at baseline showed an isolated slight increase in 3‐hydroxyglutarate with no glutarate excretion detectable. Dry blood spot free carnitine was normal on several occasions and did not increase as much as her urinary free carnitine after commencement of supplementation (free carnitine 44.4 μmol/L after 48 hours, reference range 15.5‐46.7, no increase in absolute C5DC concentration, normal C5DC/C10‐OH ratio, essentially normal ACP). During follow‐up, her dried blood spot ACP did only intermittently show a slightly raised C5DC/C10‐OH ratio at around 0.1 μmol/L (reference range 0.00‐0.07). Her biochemical results are summarized in Table 2. Plasma amino acids remained normal. Confirmation of the diagnosis was achieved by analysis of GCDH enzyme activity in cultured skin fibroblasts which was reduced to 20% of normal at 37°C with a further reduction in function at 41°C, consistent with this diagnosis (Dr Simon Olpin, Department of Clinical Chemistry, Sheffield Children's Hospital, Sheffield, UK). Her brother showed no biochemical abnormality and analysis of *GCDH* gene was not consistent with being affected.

**TABLE 2:** Blood and urine biochemical test results before and after 48 hours of carnitine supplementation

Parameter	Result	Unit	Reference range
Urine glutaric acid	Isolated slight increase in 3‐hydroxyglutarate	μmol/mmol creat.	<10
3‐Hydroxyglutarate (baseline)	(>8)^a^	μmol/mmol creat.	<8
Pre carnitine supplementation			
Blood free carnitine	30	μmol/L	15.5‐46.7
Urine free carnitine	1.99	μmol/mmol creat.	2.45‐125.5
Urinary C5DC	6.8	μmol/mmol creat.	<1.7
Post carnitine supplementation			
Blood free carnitine	44	μmol/L	15.5‐46.7
Urine free carnitine	411	μmol/mmol creat.	2.45‐125.5
Urinary C5DC	8.2	μmol/mmol creat.	<1.7
Fibroblast GCDH enzyme activity	20%	Of normal	

^a^ Estimate; not analyzed post carnitine supplementation.

Given her initial blood ACP was not indicative of carnitine deficiency, urine analysis for acylcarnitines, incl. C5DC was performed as it is the most sensitive biochemical test available. It should be considered in cases where the diagnosis is suspected but other biochemical tests do not contribute to diagnosis.^2^

Treatment was initiated with trihexyphenidyl to attenuate her dyskinesia with limited effect and she was subsequently commenced on Gabapentin with some improvement. Dietary management was implemented, including the reduction of natural protein intake and the implementation of an “unwell” regimen as well as L‐carnitine supplementation (100 mg/kg/day). Thus far, there has been limited clinical improvement in the patient and the likely benefit from ongoing management will be to prevent deterioration, particularly during intercurrent febrile illness.

## 2 DISCUSSION

Glutaric aciduria type 1 is an autosomal recessive inherited organic aciduria due to a defect in metabolism of lysine, hydroxylysine and tryptophan.^3^ The *GCDH* gene is over 7 kB on Chromosome 19p13.2 with 11 exons and there are over 150 disease‐causing mutations in the *GCDH* gene identified.^4^ There are multiple ethnic groups where prevalence of pathogenic variants is very high with a carrier frequency of up to 1:10 in the (a) Amish community, (b) Oji Cree First Nation, (c) Irish Travellers, and (d) Lumbee Tribe of North Carolina^5^; however, as this case demonstrates GA‐1 is not limited solely to these groups and should be considered when the clinical picture is suggestive, as disease‐causing pathogenic variants are found worldwide.^6^, ^7^

The classical clinical manifestation is microencephalic macrocephaly which leads to stretching of bridging veins explaining the increased incidence of subdural hemorrhage in these patients.^8^ Our case is unusual in that her OFC was not enlarged, with her most recent being on the 11th centile. Certain case series for specific populations have found macrocephaly to be present in only a subgroup of patients, in a Swedish‐Norwegian case series of 12 patients only 3 showed macrocephaly, however within the same cohort of the 10 patients that had CT/MRI brain, 7 showed evidence of temporal hypoplasia.^9^ The presence of macrocephaly appears to significantly vary with population, as in one case series of 77 patients in the United States,^8^ in patients from the Amish community with a recorded OFC, 52% (19/36) were at or above the 95th centile, whereas in patients from the general population 90% (27/30) showed an OFC >95th centile. Hydrocephalus was more common in the general population cohort. Some patients with an OFC of 20th to 25th centile were recorded but the vast majority in both groups was above the 50th centile. While macrocephaly is expected in patients with GA‐1, its absence does not out‐rule the diagnosis.

Its natural history when untreated may be characterized by onset of neurological disease during brain maturation following an acute encephalopathic crisis, which is often precipitated by an intercurrent febrile illness, gastroenteritis, surgical intervention or immunizations.^10^ Catabolism in the postnatal transition period may contribute to manifestations of neurological impairment and our case had poor feeding in the neonatal period which may have contributed to her phenotype.

The typical intracranial injury is bilateral striatal injury, which in turn can lead to a complex movement disorder with dystonia superimposed on axial hypotonia.^10^ There is a risk of severe dystonic episodes with potential status dystonicus leading to hyperthermia and rhabdomyolysis. Onset may be quite insidious with the onset of a complex movement disorder without an acute encephalopathic crisis.^11^ Acute crisis and insidious presentation may have comparable underlying mechanisms but may differ with regard to the time line of their presentation; in both cases, catabolism may contribute to the neurological injury.^12^ In a study of 22 patients with GA‐1 showing striatal changes on MRI, 11 patients had a history of acute encephalopathic crises while 10 had insidious onset.^11^ Detailed radiological analysis showed differences in pattern between insidious and acute onset patients with the former tending to demonstrate dorsolateral putaminal lesions (8 of 10) while all acute onset patients showed extensive striatal lesions. Importantly those who had initial insidious onset GA‐1 followed by an acute encephalopathic crisis showed superimposed extensive striatal injury on their preexisting dorsolateral putaminal changes.

### 2.1 Pathophysiology

**FIGURE 2:**
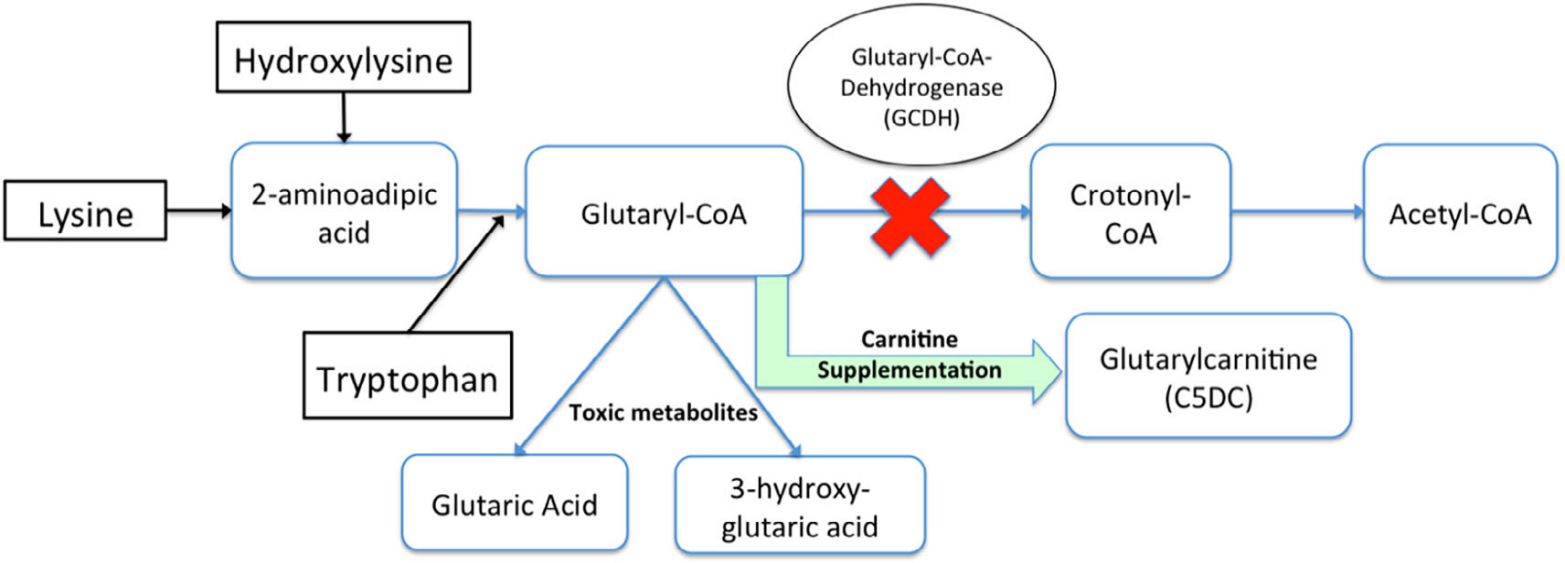
Lysine/hydroxylysine/tryptophan metabolic pathway (simplified) including proposed therapeutic effect of carnitine supplementation

GA‐1 is an organic aciduria that results from dysfunction or absence of glutaryl‐CoA dehydrogenase that is part of the pathway for metabolism of lysine, hydroxylysine, and tryptophan (Figure 2). Interruption of this leads to an inability to produce Acetyl‐CoA, which is further metabolized before partaking in the Krebs cycle.^13^ As with most organic acidurias and aminoacidopathies, the underlying pathophysiology includes intoxication with by‐products, namely glutaric acid and 3‐hydroxyglutaric acid,^10^ leading to enzyme inhibition, substrate deficiencies and energy deficiency. These accumulate in the urine, blood and CSF with particular accumulation in the CSF due to limited blood brain barrier permeability leading to their more pronounced neurotoxic effects and GA‐1 being classified as a cerebral organic aciduria.^13^ In addition to the formation of toxic metabolites, there is also secondary carnitine depletion. Supplementation with carnitine may also have a beneficial effect in metabolizing glutaryl‐CoA to C5DC which increases available Coenzyme A.^13^

Two biochemical—but clinically indistinguishable—diagnostic subgroups of GA‐I exist based on urinary excretion of glutaric acid, low excretors, and high excretors, both have similar risk of striatal injury and the distinction is not useful in distinguishing between mild and severe phenotypes. Its relevance is for the sensitivity of investigation, for example, in high excretor risk populations, the number of false negatives is low for example, Irish travellers and Amish but for low excretor populations such as the Oji Cree First nation there were a high number of false negatives.^14^, ^15^ The precise reason for this variability in excretion load remains unknown.^16^ A number of *GCDH* pathogenic variants have been reported which are more prevalent in low excretors, such as the p.Met405Val (M405V) variant in African Americans.^17^ The other most commonly reported low excretor associated mutations are p.Arg227Pro and p.Val400Met.^17^, ^18^, ^19^, ^20^ Other variants have been noted in the context of low excretor patients; however, the strength of this association is less certain. In one paper showing 30 novel mutations, it was noted that p.Ala293Thr may potentially be associated with the low excretor phenotype as well as p.Gly178Arg and p.Arg88Ser; however, these have since been observed in a high excretor phenotype.^20^, ^21^ A case series of 14 South African children showed almost all had at least one p.Ala293Thr mutation (12 of the 13 with genetic results), 11 of these 13 were homozygous for this mutation of whom 4 were classified as low excretors,.^7^ This finding suggests that, for many variants, genotype is indicative rather than definitive of excretor type. Other variants which have been reported in the presence of a low excretor phenotype include p.Pro286Ser,^19^ p.Leu221Pro,^1^ p.Arg313Trp, p.Arg234Trp,^22^ p.Arg88Cys, p.Phe236Leu, p.Ser259Pro,^23^ p.Ser119Leu, p.Ala195Thr, and p.Tyr155His.^4^ The literature currently supports the assertion that p.Arg227Pro and p.Val400Met and p.Met405Val are strongly associated with the low excretor phenotype, for many other variants, further data are needed. Interpretation is further complicated by compound heterozygosity, for example, even p.Arg227Pro when coupled with p.Arg402Trp has shown a severe high excretor phenotype.^4^

The known pathogenic variant observed in our patient; *GCDH* variant c.281G > A;pArg94Gln, has been described in the literature as being associated with a low excretor phenotype in a case series of 17 Indian patients with GA‐1 with 1 patient having this variant.^1^ This patient was compound heterozygous in the presence of an additional mutation c.662 T > C(p.Leu221Pro).^1^ Experimental studies on Arg94Gln have shown the a significantly reduced *K*_cat_ (turnover of substrate per unit time) of GCDH to 2% to 3% of wild type GCDH, this reduction is similar to the previously known disease‐causing mutation p.Arg94Gly.^24^ Based on their analysis the authors conclude that Arg94 does not make a major contribution to glutaryl‐CoA binding but that its electrical field may facilitate deprotonation of substrate.^24^

Research has been performed in more sensitive methods for newborn screening,^25^ these primarily focus on accurately detecting raised C5DC concentrations. In the Republic of Ireland GA‐1 screening (by measurement of C5DC) was added to newborn screening in December 2018 and is not expected to detect low excretors but will detect the more prevalent high excretor form in the Irish cohort.

### 2.2 Investigations

Radiological investigations hold a prominent place in the literature on GA‐1; however, interpretation of MRI findings is complex due to their wide spectrum, no findings are pathognomonic and none replace the need for a full metabolic investigation.^26^ Numerous studies have been performed on the pattern of MRI changes in patients with GA‐1. The commonest findings are striatal abnormalities, primarily of the putamen, alteration to frontotemporal CSF spaces and sub‐ependymal pseudocysts. Other deep gray matter structures such as isolated palladial or dentate nuclei lesions have sporadically been described in addition to white matter changes such as delayed myelination.^26^, ^27^, ^28^, ^29^

The use of statistical analysis has shown promise with regard to bringing order to radiological findings. One study analyzed the correlation of multiple MRI findings with each other as well as to the clinical outcome for 180 patients with a confirmed diagnosis of GA‐1.^26^ Their findings suggested the two strongest predictors of poor neurological outcome were T2 putamen hyperintensity/atrophy as well as significantly dilated ventricles. MRI findings tended to cluster together into discrete groups with cortical changes often seen with increased CSF spaces and ventriculomegaly while caudate and putamen lesions tended to occur together. Changes in gray matter tend to be more strongly correlated with disease severity than white matter changes.^26^, ^27^ Volume loss, which was present in our case, has been variably referred to as frontotemporal atrophy or hypoplasia in the literature. Historically, the term frontotemporal atrophy has been used but given radiological evidence of volume loss in the neonatal period including preterm infants and on late gestation, fetal MRI has led to advocacy for the use of the term hypoplasia.^28^ Frontotemporal hypoplasia may resolve with the onset of treatment in patients who are diagnosed in the neonatal period.^28^ The evidence now suggests that temporal hypoplasia, widening Sylvian fissures and delayed may represent intrauterine pathology while white matter changes tend to progress with age.^11^, ^26^, ^28^, ^30^ Striatal lesions, either after an acute encephalopathic crisis or during insidious onset appear to develop in infancy and early childhood (up to 3 years).^26^ For our patient the clinical manifestations appeared more related to her striatal changes as opposed to her volume loss. In our case, the use of a genetic dystonia panel highlighted one known pathogenic variant in addition to the presence of a VUS. Genetic testing could not therefore be considered to be diagnostic but rather pointed toward the diagnosis of GA‐1. This highlights both the benefits and risks of genetic analysis in the context of metabolic disease in the presence of a VUS. Genetic testing can highlight potential pathology that requires further investigation but genetic testing alone is insufficient Appropriate biochemical investigations, targeted toward the specific suspected pathology, ultimately determine the diagnosis.

The gold standard investigation is GCDH enzyme analysis of skin fibroblasts or alternatively genetic analysis for known disease‐causing variants.^10^, ^31^, ^32^ Enzyme analysis is crucial in demonstrating the pathogenicity of detected VUS, as was the case here, this enzymatic analysis is a highly refined investigation performed at specialist laboratories. Urine organic acids are useful in diagnosis of high excretors showing raised glutaric and 3‐hydroxy glutaric acids; however, a normal or essentially normal urine organic acid pattern does not exclude GA‐1 even when repeated multiple times. Urinary excretion of C5DC is a sensitive specialized test to be performed in suspected cases of atypical GA‐1.^2^

### 2.3 Management

The mainstay of treatment once diagnosis is made is to limit further neurological injury and alleviate current symptoms.^5^, ^32^, ^33^, ^34^ Dietary management is of primary importance with an adjusted diet needed to limit intake of lysine/hydroxylysine and tryptophan. l‐Carnitine supplementation is given to alleviate the secondary carnitine depletion associated with most organic acidurias.^35^ There is currently no evidence for neuroprotective agents such as *N*‐acetyl cysteine, topiramate, antioxidants, or phenobarbitone.^33^ Emergency management of an intercurrent illness is aimed at preventing catabolism and metabolic crisis. A sick day regimen for home management includes, for example, double dose carnitine and high carbohydrate, zero natural protein formula but lysine‐free, low tryptophan amino acid supplements. Inability to tolerate this regimen warrants admission and IV dextrose with the aim to maintain fluid, electrolyte and pH balance and to reduce catabolism. These rules are to be followed during any period of high stress including immunizations and surgical procedures. This applies to both high and low excretor phenotypes. Exclusion of natural protein intake is designed to be transient (eg, for 24‐48 hours) and prolonged periods of illness require careful adjustments of the patient's dietetic and medical treatment. The risk of striatal injury is most pronounced up to age 6 years when dietary controls are most strict; thereafter, there is more scope for some relaxation in some cases.^33^ Adherence to these guidelines is associated with improved neurological outcome.^5^, ^36^ The neurological sequelae are managed with baclofen or diazepam for movement and tone disorders. Anticholinergics, typically trihexphenidyl are used to alleviate dystonia with varied responses. Botulin toxin type A may also be used in the presence of hypertonicity. The outcome is generally poor if neurological findings have become manifest.

## 3 CONCLUSION

The case of GA‐1 presented here highlights a number of issues of interest both to the metabolic specialist and to the general clinician. GA‐1 is an area of ongoing research, including reports of new, potentially pathogenic variants including *GCDH* c.1082 + 2 T > G; reported here. Neuroimaging is now to be interpreted cautiously as more information becomes available on changes in imaging over the course of a patients growth and development. While diagnosis has been significantly aided by its addition to newborn screening in many countries,^37^ the existence of the low excretor phenotype coupled with a highly variable clinical presentation makes diagnosis for a small but important cohort difficult. Genetic analysis alone may be diagnostic if known disease‐causing mutations are detected, however, with the continuously growing listing of reported mutations this is far from definitive. Urine C5DC is an important test in the presence of an otherwise normal biochemical profile. Enzymatic analysis constitutes the gold standard investigation but requires highly specialized laboratory facilities that may not be readily available to all clinicians in a timely manner. For the metabolic specialist this case underscores the significant clinical, genetic and biochemical variability of the disease which warrants careful and thorough investigation when suspicion is aroused either by clinical presentation or a genetic VUS. For the general clinician this case highlights the benefit of genetic panels for specific clinical presentations but caution with interpretation of variants of uncertain significance is warranted and close consultation with a tertiary metabolic center is advised to direct further investigations as appropriate. GA‐1 should be an important differential for dystonia in a patient from any population, particularly with neuroimaging suggesting striatal injury.

## CONFLICT OF INTEREST

The authors declare no conflict of interest.

## AUTHOR CONTRIBUTIONS

**Jason Foran** and **Niamh McSweeney**: Involved in the initial evaluation, investigation, preparation and editing of the manuscript. **Ellen Crushell** and **Ina Knerr**: Contributed to the management of the patient and editing and preparation of the manuscript. **Michael Moore**: Radiological evaluation and contribution of appropriate images.

## PATIENT CONSENT STATEMENT

Informed consent for investigation and presentation were obtained from the patient's parents and the case study was performed in compliance with applicable regulations, standards, and ethical guidelines.
